# Improving Prevention of Mother-to-Child Transmission of HIV Care and Related Services in Eastern Rwanda

**DOI:** 10.1371/journal.pmed.1000302

**Published:** 2010-07-20

**Authors:** Younsook Lim, Jim Yong Kim, Michael Rich, Sara Stulac, Jean Bosco Niyonzima, Mary C. Smith Fawzi, Rose Gahire, Martha Mukaminega, Marya Getchell, Curtis W. Peterson, Paul E. Farmer, Agnès Binagwaho

**Affiliations:** 1 Department of Pediatrics, Dartmouth Medical School, Hanover, New Hampshire, United States of America; 2 Dartmouth Medical School, Hanover, New Hampshire, United States of America; 3 Partners in Health-Rwanda, Kigali, Rwanda; 4 Brigham and Women's Hospital, Boston, Massachusetts, United States of America; 5 Harvard Medical School, Boston, Massachusetts, United States of America; 6 Division of Global Health Equity, Brigham and Women's Hospital, Boston, Massachusetts, United States of America; 7 Head of Infectious Clinic, Rwinkwavu Hospital, Rwinkwavu, Rwanda; 8 Partners In Health, Boston, Massachusetts, United States of America; 9 Department of Global Health and Social Medicine, Harvard Medical School, Boston, Massachusetts, United States of America; 10 AIDS HealthCare Foundation, Kigali, Rwanda; 11 Pediatric Care and Treatment, Elizabeth Glaser Pediatric AIDS Foundation, Kigali, Rwanda; 12 François-Xavier Bagnoud Center for Health and Human Rights, Harvard School of Public Health, Boston, Massachusetts, United States of America; 13 Ministry of Health, Kigali, Rwanda

## Abstract

Younsook Lim and colleagues describe the Rwanda Learning Collaborative on Child Health, which aimed to improve and extend the impact of prevention of mother-to-child transmission of HIV/AIDS.

**Summary Points**

- Comprehensive prevention of mother-to-child transmission of HIV/AIDS (PMTCT) care requires following mothers and infants longitudinally to ensure quality care is delivered.
- Targeted disease-specific programs can be designed to have an impact on related nontargeted services to increase the health value delivered to families.
- Quality improvement methods can be effective tools for engaging local health workers and targeting investment of limited resources to maximize improvement of services.
- A learning collaborative approach is one method that can garner cooperation between nongovernmental organizations and governments to improve coordination of services and strengthen health systems.

## Introduction

A recent World Health Organization (WHO) report found a complex array of positive and negative impacts of global health initiatives (GHIs) on existing health systems [1]. The report's examination of published studies and both quantitative and qualitative data collected in 15 countries demonstrated the need for programs proactively designed to have positive effects on “nontargeted” health services. Here, we describe an effort that was designed to improve the quality and extend the impact of prevention of mother-to-child transmission of HIV/AIDS (PMTCT) care in a manner that benefited multiple areas of clinical care.

The 2006 WHO treatment guidelines for PMTCT emphasize that efforts to decrease transmission must be based on the delivery of broad maternal and child health services. Such approaches can reduce transmission rates to less than 2% [2]. Studies to improve PMTCT suggest that in order for interventions to be most effective, they should be delivered via decentralized, community-based, longitudinal programs [3],[4].

In recent years, the percentage of HIV-positive pregnant women living in low- and middle-income countries who are receiving antiretroviral medication to prevent transmission to their infants has increased: from 10% in 2004 to 45% in 2008 [5]. However, this statistic includes women who received a single dose of nevirapine at the onset of labor, indicating that coverage rates for women receiving PMTCT care according to WHO treatment guidelines are lower.

## The Rwanda Learning Collaborative

The Rwanda Learning Collaborative on Child Health (RLC) was conceived as a service delivery component of the Joint Learning Initiative on Children and HIV/AIDS (JLICA). The project sought to increase access to and the quality of PMTCT services in the Eastern Province of Rwanda using a learning collaborative model. This method focuses on peer-to-peer learning through periodic “learning sessions,” meetings at which participants convene to discuss improvement of selected indicators. The approach allows for multiple improvement ideas to be simultaneously tested and evaluated [6].

Although using the learning collaborative model to spread best clinical practices in developed countries has had mixed results [7],[8], the ability of this method to engage local leadership and health care practitioners to implement changes in a short time frame is an acknowledged strength [9]. Similar approaches focusing on systems analysis and measurement of targeted interventions have been used successfully in resource-poor countries [10]–[12], but there has been disagreement over the provision of additional resources in such instances [13],[14]. The RLC worked with the Rwandan Ministry of Health (MOH), the Rwandan Treatment and Research AIDS Center (TRAC), and partner nongovernmental organizations (NGOs) to provide technical and financial support to participating health centers [15]. Improvement ideas requiring additional resources received funding from the RLC on a case-by-case basis.

The RLC employed all key elements of a learning collaborative. Four learning sessions were held between June 2007 and April 2009; these were one-day meetings where health center participants and experts from TRAC and NGO partners reviewed health center progress and technical information. “Action periods” occurred between learning sessions. During these periods, health center staff tested improvement ideas, which were evaluated through the use of standard time plots charting an indicator (for example, number of women receiving antenatal care) against months. When testing ideas, health center staff applied a quality improvement approach known as a plan-do-study-act (PDSA) cycle that focuses on executing and evaluating improvement ideas on a small scale by a team of health providers. The approach has a specific structure: planning an improvement idea, testing it (“do”), studying the results, and acting to make additional changes based on the results [6].

Rwanda is moving toward a decentralized health care system that provides HIV/AIDS and PMTCT services at the health center level. The country has nationalized health insurance, a structured community health worker (CHW) system, and its government oversees all NGO activities [16]. Rwanda also has an opt-out HIV testing policy for all pregnant women and their partners. National HIV prevalence was estimated to be 3% in 2007 [17]. Seventeen health centers participated in the RLC, each supported in part by one of four NGOs. The largest health center served a population of roughly 57,000 people, the smallest slightly over 12,000. The team at each health center comprised an antenatal clinic (ANC) nurse and the head nurse.

To facilitate communication between health centers, a field team of two Rwandan professionals trained in the learning collaborative method provided technical support. This team visited health centers bi-weekly to assist with the development, implementation, and assessment of PDSA cycles, and collected data on selected indicators. The field team received logistical and administrative support from the François-Xavier Bagnoud Center for Health and Human Rights at Harvard University.

**Figure 1:**
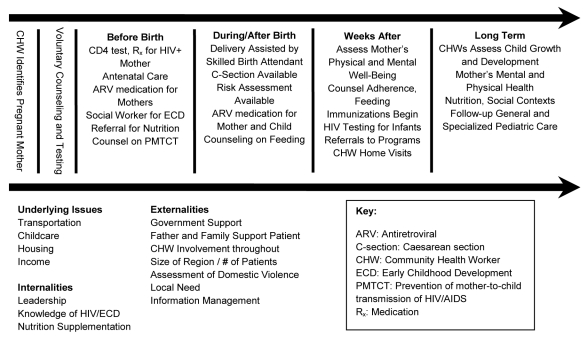
Value chain analysis as applied to PMTCT.

Experts from the JLICA and TRAC convened in Kigali in March 2007 to conduct a value chain analysis of PMTCT care (see Figure 1) [18]. The analysis divided PMTCT care into a series of steps and identified interventions that added “value” to PMTCT care at every stage. Understanding how to reach the maximum health outcome achievable by delivery of services to mothers and infants affected by HIV was the goal of this exercise. Important components of care were identified: access to PMTCT services; completion of PMTCT regimen; support of infant feeding methods to reduce postnatal transmission; access to co-trimoxazole and bed nets for infants; monitoring for early childhood development (ECD) of infants; and completion of infant immunization regimens.

## Outcomes in Improving Care

All 17 health centers carried out improvement activities in target areas for the duration of the project. Thirty-four unique ideas covering all areas of improvement were tested. Increasing health center attendance through provision of material incentives, expanding access to services through outreach sites, and strengthening links between patients and health care providers through CHWs were among the many ideas health centers explored. Although most health centers reported notable improvements during the project, these numbers are not amenable to standard tests of statistical significance because of the underlying assumptions inherent in the statistical methods used in quality improvement.

### Increasing PMTCT Coverage through Antenatal Care

Six months prior to the RLC, participating health centers averaged 20% (*n* = 283/month) first trimester ANC attendance (percent attendance = reported monthly attendance/expected monthly attendance. Expected monthly attendance is based on the total fertility rate for women of childbearing age (15–49 y) in each health center's catchment area population). During the final 8 mo of the RLC, health centers reported an average of 41% first trimester ANC attendance (*n* = 613/month). Eleven health centers tested paying CHWs for each pregnant woman brought to clinic; others offered material incentives directly to pregnant women to attend or conducted community education on ANC. Health centers reported varying increases in clinic attendance during the months of testing. In the last 8 mo, all health centers agreed to offer women incentives at their first trimester ANC visit, to have nurses conduct weekly community education sessions, and to pay CHWs a monthly stipend to refer all pregnant women in their catchment to the health center.

### Securing Continuity of PMTCT Care

At the outset of the project, only four health centers used a devoted registry to track women through PMTCT care. By the end of the project, 11 health centers used a separate PMTCT registry for tracking HIV-positive women and 15 reported 95% of HIV-positive pregnant women receiving PMTCT services (*n* = 116).

National data from 2007 indicate that 87% of women attending their first ANC visit learned their HIV status [17]. In the fall of 2007, 12 health centers identified 174 HIV-positive women who had failed to continue attending clinic appointments for PMTCT care. Women often exited PMTCT care when referred to a hospital. Multiple interventions were tested to address this problem: drawing blood at the health center and sending the specimen for CD4 analysis, verifying with hospital staff that referred women kept their appointments to receive ARVs, and conducting home visits by nurses to find women who missed their appointments.

### Infant Feeding

Rwanda's national policy to reduce postnatal HIV transmission follows WHO guidelines, which recommend that HIV-positive mothers either use infant formula if acceptable, feasible, affordable, sustainable, and safe (AFASS), or exclusively breastfeed and wean at 6 mo [19]. Six health centers provided infant formula to HIV-positive mothers, two referred HIV-positive women to a hospital for infant formula, and nine counseled women to breastfeed exclusively and wean at 6 mo. A major barrier to weaning was lack of appropriate replacement foods. Here, the addition of resources in the form of supplemental food was critical to improvement. Supporting NGOs and a consortium of international agencies were able to provide SOSOMA (a mixture of sorghum, soya, and maize) to HIV-exposed infants at 6 mo at four health centers. The RLC provided funding for six additional health centers to distribute SOSOMA during the last 8 mo of the project. All mothers whose children were receiving SOSOMA received counseling on weaning (*n* = 102). Health centers confirmed weaning through breast exams, home visits to mothers in the community, or observation of mother-infant interaction during clinic visits.

### Co-trimoxazole Prophylaxis

Eleven health centers initially offered co-trimoxazole prophylaxis. Six health centers reported that up to half of children identified to receive prophylaxis had not returned to the clinic for several months. By the last 8 mo of the project, 15 health centers offered co-trimoxazole prophylaxis. At ten health centers reporting consistent monthly data, an average of 98% of HIV-exposed infants received prophylaxis (*n* = 277/month).

The largest obstacle reported was traveling long distances to health centers in order to receive the medication. In one testing cycle, co-trimoxazole was given to HIV-exposed infants on the same day each month, coupled with other services such as feeding counseling, and coinciding with market days when women were likely to be near the health center. The first health center to test this idea reported an increase in coverage from 40% to 100%. This outcome was shared at the second learning session and the intervention was implemented at ten additional health centers.

### Early Childhood Development

Monitoring ECD was considered an important component of comprehensive PMTCT care. However, the field team observed that ECD screening at health centers consisted of measuring only height and weight. The RLC developed an ECD tool using “Care for Development” guidelines from the WHO's Integrated Management of Childhood Illness to increase implementation of comprehensive ECD monitoring in the community. Training on ECD and use of the ECD tool was organized for 34 nurses from all health centers. These nurses in turn trained CHWs at their respective health centers on how to use the tool during home visits to counsel caregivers on ECD. Across ten health centers, nurses reported that 962 CHWs received training by the end of the project.

### Vaccinations and Bed Nets

From July to November 2007, vaccination coverage averaged 83%. Vaccination completion increased to an average of 90% during the last 8 mo of the project by increasing the number of outreach vaccination sites and having CHWs visit children who missed appointments. Health centers were able to increase bed net distribution from 80% to 94% in the first 10 mo of the project by improving distribution of bed nets to outreach sites until a nationwide stock out of bed nets.

## Utility of the Learning Collaborative Model

The experience of the RLC suggests that the learning collaborative model can have a positive impact not only on the quality of targeted areas of care, but also on the capacity of health centers to more effectively deliver nontargeted services. This systems-based approach was useful in encouraging problem solving in a local context, assisting in the determination of where resources were needed, and encouraging demand from those who utilize services.

### Strengthening Health Systems

A questionnaire at the final learning session was completed by 28 out of 29 learning session attendees from participating health centers. Among those responding, 71% found the project to be different in significant ways from others conducted by the MOH or other NGOs; 91% found using time plots to be helpful with 82% believing they would continue to use them; and 77% found using PDSA cycles to be a helpful tool in making improvements, with 70% believing they would continue to use them. Investments in human resources such as this, which empower staff to take initiative in identifying the need for improvements and to act to remedy them, represent a worthwhile development of local capacity.

The supplemental funding of health center budgets by the RLC was relatively small. Cloth wraps for women cost FRW1,500 (US$2.64) each, a CHW monthly stipend was FRW2,500 (US$4.40), and a month of supplemental food for weaning was FRW10,500 (US$18.50) per infant. These small investments were often followed by substantial improvements, suggesting that quality improvement projects may be effective tools not only for improving service delivery but for leveraging funding.

### Developing Local Solutions and Improving Nontargeted Services

Addressing barriers at the local level improved health services for all women regardless of HIV status. For example, although not an explicit focus of the project, women delivering at participating health centers increased from 56% in 2007 to 72% in 2008, which is higher than the 2008 national average of 45% [20]. This outcome resulted from an observation made by health center staff that deliveries also needed improvement, rather than a target set at the beginning of the project.

Through the RLC, local health center staff viewed the improvement of immunization services, bed net distribution, and early childhood screening, which are not traditionally included in programs targeting PMTCT, as synergistic components of delivering care to affected women and children rather than as competing programs.

### Challenges and Next Steps

Comprehensive PMTCT services involve multiple systems of care. Coordination between several governmental agencies and supporting partners at health centers at times posed obstacles to a unified approach to PMTCT. For example, in an effort to train community health workers on reproductive health as part of raising community awareness, the RLC had to obtain approval from: the Community Health Desk, the Maternal and Child Health Unit, TRAC, the health centers' supporting NGOs, and each health center. The RLC was able to work within this complex system of stakeholders largely owing to the MOH's active engagement. Recommendations made by health center staff or the support team that required substantive change to current procedures were communicated to TRAC and the MOH for support. Thus the Rwandan government was invaluable in providing leadership and fostering cooperation.

While learning collaborative methods such as the RLC offer an effective structure for disseminating best practices and sharing solutions at the grass-roots level, this project required significant external technical support to make effective improvements. In other settings, this requirement may impose a barrier to the outcomes that can be achieved by this approach. The addition of financial resources to overcome specific barriers also poses challenges in resource-poor countries where governments and NGOs may be working with limited budgets.

As the influence of global health initiatives (GHIs) continues to grow, the architects of programs targeting specific diseases or areas of care can commit themselves to considering the impact their interventions can have on health systems and outcomes beyond their immediate focus. Quality improvement approaches could be employed as effective organizing tools under active government guidance with the cooperation of NGOs to target investment of resources and strengthen health systems.

## Abbreviations

ANC: antenatal clinic
CHW: community health worker
ECD: early childhood development
MOH: Rwandan Ministry of Health
NGO: nongovernmental organization
PDSA: plan-do-study-act
PMTCT: prevention of mother-to-child transmission of HIV/AIDS
RLC: Rwanda Learning Collaborative on Child Health
TRAC: Rwandan Treatment and Research AIDS Center
WHO: World Health Organization
